# Increased alcohol intake is associated with radiographic severity of knee and hand osteoarthritis in men

**DOI:** 10.1038/s41598-024-63559-x

**Published:** 2024-06-02

**Authors:** Haimuzi Xu, Ji-Hyoun Kang, Sung-Eun Choi, Dong-Jin Park, Sun-Seog Kweon, Young-Hoon Lee, Hye-Yeon Kim, Jung-Kil Lee, Min-Ho Shin, Shin-Seok Lee

**Affiliations:** 1 Division of Rheumatology, Department of Internal Medicine, Chonnam National University Medical School & Hospital, 42 Jebong-Ro, Dong-Gu, Gwangju, 61469 Republic of Korea; 2 Department of Rheumatology and Immunology, Affiliated Hospital of North Sichuan Medical College, Nanchong, Sichuan Province China; 3 Department of Preventive Medicine, Chonnam National University Medical School, 160 Baekseo-Ro, Dong-Gu, Gwangju, 61469 Republic of Korea; 4 Jeonnam Regional Cancer Center, Chonnam National University Hwasun Hospital, Hwasun, Republic of Korea; 5 Department of Preventive Medicine & Institute of Wonkwang Medical Science, Wonkwang University College of Medicine, Iksan, Republic of Korea; 6 Department of Neurosurgery, Chonnam National University Medical School and Hospital, Gwangju, Republic of Korea

**Keywords:** Osteoarthritis, Alcohol, Knee, Hand, Rheumatology, Risk factors

## Abstract

Observational studies have shown controversial associations between alcohol intake and radiographic osteoarthritis (OA). This study investigated whether this association was causal using a Mendelian randomization (MR) study in a population-based cohort in Korean. The study enrolled 2429 subjects (1058 men, 1371 women) from the Dong-gu Study. X-rays of the hand and knee joints were scored using a semi-quantitative grading system to calculate the total score of the hand and knee joints. ALDH2 rs671 genotyping was performed by high-resolution melting analysis. MR instrumental variable analysis and observational multivariable regression analysis were used to estimate the association between genetically predicted alcohol intake and the radiographic severity of OA. Subjects with the G/G genotype had a higher current alcohol intake than those with the G/A and A/A genotypes in both men and women (all P < 0.001). Men with the G/G genotype had higher total knee (P < 0.001) and hand scores (P = 0.042) compared to those with the G/A and A/A genotypes after adjusting for age and body mass index, but not in women. In the observational multivariable regression analysis, each alcohol drink per day in men was associated with increased knee (P = 0.001) and hand joint scores (P = 0.013) after adjustment, but not in women. In our MR analysis, utilizing ALDH2 rs671 genotypes as instrumental variables for alcohol consumption, has shown a significant link between each additional daily alcohol drink and increased radiographic joint severity in men.

## Introduction

Osteoarthritis (OA) is a common degenerative joint disease that affects various joints, including the hand, knee, and hip. It is characterized by articular cartilage damage, osteophyte formation, and synovial inflammation, leading to clinical symptoms like pain, stiffness, joint swelling, limited movement, and deformity^1^. The risk factors for OA include age, gender, obesity, joint injury or trauma, and genetic predisposition. Additionally, alcohol consumption has been suggested as a potential risk factor for OA^2^.

Previous studies have investigated the relationship between alcohol intake and OA, yielding conflicting results when considering different joints. Notably, in hand OA, moderate alcohol consumption showed a positive association with radiographic severity^3^, while other studies reported either a negative correlation^4,5^ or no significant link^6,7^ between alcohol consumption and symptomatic and radiographic hand OA. Similarly, in knee OA, alcohol consumption demonstrated a significant association with radiographic knee OA in certain studies^8^, but others did not find such a connection^7,9^. On the contrary, alcohol intake did not appear to be a risk factor for the development of hip OA^10^, nor was it associated with radiographic hip OA^11^. It is essential to acknowledge that the observed association between alcohol intake and OA in these studies may be subject to bias, which could result from small sample sizes, alcohol intake not being the primary variable of interest, inadequate or inappropriate use of radiographic examinations, and evaluating individual joints without considering the entire joint complex. As a result, the direct impact of alcohol consumption on the radiographic severity of OA remains uncertain.

The enzyme aldehyde dehydrogenase-2 (ALDH2), located on chromosome 12q24, plays a significant role in ethanol metabolism. After alcohol ingestion, ethanol is converted to acetaldehyde by alcohol dehydrogenase, and ALDH2 is responsible for further catalyzing acetaldehyde to acetic acid. Genetic variations due to point mutations lead to different ALDH2 alleles: G/G (wildtype), G/A (inactive heterozygotes), and A/A (inactive homozygotes), resulting in varying enzymatic activity of ALDH2. Individuals with G/A and A/A genotypes experience an accumulation of acetaldehyde in the body, leading to physiological adverse reactions such as facial flushing^12^. Consequently, those with G/A and A/A genotypes tend to consume less alcohol than those with the G/G genotype. The ALDH2 rs671 polymorphism can be used as an instrumental variable for alcohol consumption^13^.

Genetic variants, unlike environmental factors such as alcohol consumption, are not influenced by confounders like socioeconomic status, behavioral risk factors, and psychological variables, which can affect the association between alcohol and OA. Using the Mendelian randomization (MR) approach with ALDH2 rs671 as an instrumental variable can help establish a causal relationship between alcohol intake and OA. However, no studies have used MR to investigate the relationship between alcohol intake and radiographic OA. Additionally, previous studies on this association have relied on the low-accuracy Kellgren–Lawrence (K–L) grading method. Therefore, we conducted an MR study using a novel, semi-quantitative grading system in a large, population-based cohort to investigate the association between alcohol consumption and radiographic features of hand and knee OA.

## Methods

### Study design and population

Subjects were recruited from the Dong-gu study, a population-based cohort in the Dong-gu area of Gwangju Metropolitan City, Republic of Korea. Between May 2007 and July 2010, 34,040 eligible residents (15,552 men and 18,488 women) aged 50 years and over were identified. Of these, 9260 participants (3711 men and 5549 women) were enrolled at the baseline survey, resulting in a participation rate of 27.2% (23.9% for men and 30.0% for women)^14^. In 2009, imaging studies were conducted on 2516 individuals, and full sets of knee and hand joint radiographs were available for 2489 individuals. After excluding 51 subjects with a history of knee amputation or total knee arthroplasty, 8 with missing data on drinking status, and 1 with missing data on rs671 genotype, a total of 2429 subjects were included in the final analysis of this study. Prior to participation, all subjects provided written informed consent, and the Institutional Review Board of Chonnam National University Hospital (no. CNUH-2019-336) approved the study. The research was conducted following the principles of the Declaration of Helsinki and adhered to the guidelines of Good Clinical Practice.

### Covariates

Information regarding age, gender, body mass index (BMI), alcohol consumption, education, and physical activities was collected using a standardized questionnaire. BMI was calculated as the weight in kilograms divided by the height in meters squared. Alcohol consumption was categorized into two groups: current drinkers and non-drinkers, based on their alcohol intake within the past 12 months. For current alcohol drinkers, the average number of drinks per day was estimated by considering both the frequency and quantity of alcohol consumed within the past 12 months. In addition, the average number of drinks per day was divided into two groups: less than one drink and one or more drinks per day. In this study, one drink of daily alcohol consumption was considered equivalent to the intake of 10 g of ethanol.

### Radiographic features

Anteroposterior X-rays of the hands and knees were taken while the participants were in a standing position. Two independent observers evaluated the X-ray images using a previously described semi-quantitative grading system^15,16^. This system assessed the radiographic features of the hand, including the distal interphalangeal joint, proximal interphalangeal joint, carpometacarpal joint, interphalangeal joint of the thumb, and naviculotrapezial joint. For the knee, the grading system evaluated the medial and lateral compartments, as well as the tibial and femoral components. To calculate the total score for the hand joint (maximum score = 70), the sum of the scores from six subscales was used: osteophytes (maximum score = 22), joint space narrowing (JSN) (maximum score = 22), subchondral cysts (maximum score = 4), sclerosis (maximum score = 6), erosion (maximum score = 10), and malalignment (maximum score = 6). Similarly, the total score for the knee joint (maximum score = 42) was determined by adding the scores from four subscales: osteophytes (maximum score = 24), JSN (maximum score = 12), tibial attrition (maximum score = 2), and sclerosis (maximum score = 4).

### Genotyping of ALDH2 rs671

Genomic DNA was extracted from peripheral blood using the QIAamp DNA Blood Mini Kit (Qiagen, Valencia, CA) following the manufacturer's protocol. Genotyping of rs671 was performed using high-resolution melting (HRM) analysis with a Rotor-Gene 6000TM instrument (Corbett Research, Sydney, Australia). PCR primers were utilized to produce a 97-bp amplicon, and their sequences were as follows: forward primer, 5ʹ-ttggtggctacaagatgtcg-3ʹ, and reverse primer, 5ʹ-caggtcccacactcacagttt-3ʹ. The HRM reaction mixture consisted of 200 nM PCR primer, 1 µM SYTO 9 fluorescent dye (Invitrogen, Carlsbad, CA), 0.5 U f‐star Taq polymerase (BioFACT), and 40 ng genomic DNA in 10 µL reaction volumes. The cycling conditions commenced with denaturation at 95 °C for 5 min, followed by 40 cycles of 95 °C for 5 s and 58 °C for 30 s.

### Statistical analysis

All statistical analyses were conducted using Stata version 15 (Stata Corporation, College Station, Texas, USA). Baseline characteristics of the study population were presented as mean ± standard deviation for continuous variables and were analyzed using the t-test. Categorical variables were expressed as n (%) and tested using the Chi-square test. A multiple linear regression analysis was employed to evaluate the relationship among ALDH2 rs671 genotypes, alcohol intake, and the radiographic severity of OA, with adjustments made for age, gender, and BMI. Additionally, a MR instrumental variable analysis and observational multivariable regression analysis were used to estimate the association between genetically predicted alcohol intake and the radiographic severity of OA. In these analyses, values were presented as beta coefficients along with their corresponding 95% confidence intervals (CIs). A P value of < 0.05 was considered indicative of a statistically significant difference.

## Results

### Baseline characteristics

Table 1 presents the baseline characteristics of the study population, categorized by gender. A total of 2429 subjects were included, consisting of 1058 men and 1371 women. The mean age of all subjects was 64.0 years, with men being older than women (P < 0.001). The overall mean BMI was 24.4 kg/m^2^, with women having a higher mean BMI than men (P < 0.001). Among all subjects, 49.7% were current alcohol drinkers, with a higher proportion of current alcohol drinkers observed among men compared to women (P < 0.001). The average alcohol consumption per day for all subjects was 0.91 drinks/day, and men tended to consume significantly more alcohol per day than women (P < 0.001). Additionally, 21.7% of all subjects reported drinking one or more drinks per day, and the proportion of men who drank one or more drinks per day was significantly higher than that of women (P < 0.001).

**Table 1:** Baseline characteristics of study participants stratified by gender.

	Men	Women	P-value
Number (%)	1058 (43.6)	1371 (56.4)	
Age (years)	64.9 ± 8.2	63.3 ± 8.2	< 0.001
Body mass index (kg/m^2^)	24.0 ± 2.8	24.7 ± 3.0	< 0.001
Current alcohol intake (%)	712 (67.3)	494 (36.0)	< 0.001
Alcohol drinks (day)	1.89 ± 2.65	0.15 ± 0.60	< 0.001
Alcohol ≥ 1 drink* (day)	477 (45.1)	50 (3.7)	< 0.001
ALDH2 rs671 genotypes			0.761
G/G	737 (69.7)	967 (70.5)	
G/A	296 (28.0)	377 (27.5)	
A/A	25 (2.4)	27 (2.0)	
Knee joints			
Total score	13.68 ± 5.81	15.17 ± 7.57	< 0.001
Osteophyte score	7.21 ± 3.04	7.98 ± 4.17	< 0.001
JSN score	5.75 ± 2.43	6.27 ± 2.63	< 0.001
Tibial attrition score	0.21 ± 0.55	0.25 ± 0.61	0.082
Sclerosis score	0.51 ± 0.83	0.67 ± 0.98	< 0.001
Hand joints			
Total score	16.21 ± 5.65	17.47 ± 6.69	< 0.001
Osteophyte score	6.92 ± 2.15	6.14 ± 2.04	< 0.001
JSN score	7.85 ± 2.96	9.16 ± 3.45	< 0.001
Subchondral cyst score	0.65 ± 1.02	1.33 ± 1.35	< 0.001
Sclerosis score	0.45 ± 0.90	0.35 ± 0.80	0.003
Erosion score	0.23 ± 0.88	0.34 ± 1.04	0.004
Malalignment score	0.10 ± 0.46	0.15 ± 0.51	0.021

Unless otherwise indicated, values are mean ± standard deviation.

*One drink was assumed to contain 10 g of ethanol.

JSN, joint space narrowing.

For the radiographic scores in the knee joint, women had significantly higher total score, osteophyte score, JSN score, and sclerosis score than men (all P < 0.001). In the hand joint scores, women had higher total score (P < 0.001), JSN score (P < 0.001), subchondral cyst score (P < 0.001), erosion score (P = 0.004), and malalignment score (P = 0.021) compared to men. Conversely, men had higher osteophyte score (P < 0.001) and sclerosis score (P = 0.003) in the hand joint than women.

### Association of ALDH2 rs671 genotype with alcohol consumption

Table 2 shows the frequencies of the ALDH2 rs671 genotype in the entire population, showing that 70.2% had the G/G genotype, 27.7% had the G/A genotype, and 2.1% had the A/A genotype. The observed genotype frequency distribution was consistent with the Hardy–Weinberg equilibrium (P = 0.122). Subjects with the G/G genotype had a higher proportion of current alcohol drinkers than those with G/A and A/A genotypes (59.3% vs 26.9%, P < 0.001). Additionally, subjects with the G/G genotype consumed more alcohol per day than those with G/A and A/A genotypes (P < 0.001). Moreover, the proportion of subjects who drank one or more drinks per day was higher among those with the G/G genotype compared to those with G/A and A/A genotypes (P < 0.001).

**Table 2:** Characteristics of study participants classified by ALDH2 rs671 genotypes.

	Men			Women		
	G/G	G/A and A/A	P value	G/G	G/A and A/A	P-value
Number (%)	737 (69.7)	321 (30.3)		967 (70.5)	404 (29.5)	
Age (years)	64.6 ± 8.2	65.7 ± 8.2	0.039	63.0 ± 8.1	63.9 ± 8.5	0.063
Body mass index (kg/m^2^)	24.0 ± 2.7	24.0 ± 2.9	0.826	24.8 ± 3.0	24.7 ± 2.9	0.780
Current alcohol intake (%)	581 (78.8)	131 (40.8)	< 0.001	430 (44.5)	64 (15.8)	< 0.001
Alcohol drinks (day)	2.45 ± 2.83	0.60 ± 1.52	< 0.001	0.20 ± 0.71	0.02 ± 0.09	< 0.001
Alcohol ≥ 1 drink* (day)	423 (57.4)	54 (16.8)	< 0.001	49 (5.1)	1 (0.2)	< 0.001

Unless otherwise indicated, values are mean ± standard deviation.

*One drink was assumed to contain 10 g of ethanol.

When examining the data based on gender, men with the G/G genotype had a significantly higher proportion of current alcohol drinkers, approximately two times more than men with G/A and A/A genotypes (78.8% vs 40.8%, P < 0.001). Similarly, men with the G/G genotype consumed more alcohol per day than men with G/A and A/A genotypes (P < 0.001). The proportion of men who drank one or more drinks per day in the G/G genotype group was dramatically higher than that in men with G/A and A/A genotypes (P < 0.001). In women, the proportion of current alcohol drinkers with the G/G genotype was 44.5%, around three times higher than women with G/A and A/A genotypes (P < 0.001). Women with the G/G genotype also had higher alcohol consumption per day than those with G/A and A/A genotypes (P < 0.001). Additionally, women with the G/G genotype had a higher percentage of daily drinkers who consumed one or more drinks compared to women with G/A and A/A genotypes, (P < 0.001).

### Association of alcohol consumption with radiographic severity in the knee and hand joints

The association between alcohol intake and radiographic severity scores in the knee and hand joints is shown in Table 3. Among the total subjects, those who consumed one or more drinks per day exhibited higher scores in the knee joint for total score (P = 0.005), osteophyte score (P = 0.014), JSN score (P = 0.036), tibial attrition score (P = 0.008), and sclerosis score (P = 0.005) compared to subjects who consumed less than one drink per day after adjusting for age, gender, and BMI. In the hand joints, however, the total score did not differ significantly between the two groups, yet subjects who drank one or more drinks per day displayed higher scores in osteophyte score (P = 0.049), sclerosis score (P = 0.001), erosion score (P = 0.015), and malalignment score (P = 0.008) after adjusting for age, gender, and BMI.

**Table 3:** Association between alcohol intake and radiographic severity scores in the knee and hand joints.

	Men			Women		
	≥ 1 drinks/day	< 1 drink/day	P value	≥ 1 drink/day	< 1 drink/day	P value
Knee joints						
Total score	14.13 ± 0.25	13.31 ± 0.23	0.017	15.33 ± 0.91	15.16 ± 0.18	0.857
Osteophyte score	7.43 ± 0.13	7.03 ± 0.12	0.029	7.93 ± 0.51	7.98 ± 0.10	0.929
JSN score	5.86 ± 0.11	5.65 ± 0.10	0.153	6.49 ± 0.32	6.27 ± 0.06	0.501
Tibial attrition score	0.25 ± 0.02	0.18 ± 0.02	0.045	0.35 ± 0.08	0.25 ± 0.02	0.219
Sclerosis score	0.59 ± 0.04	0.44 ± 0.03	0.003	0.57 ± 0.13	0.67 ± 0.02	0.414
Hand joints						
Total score	16.44 ± 0.23	16.01 ± 0.21	0.165	17.26 ± 0.74	17.48 ± 0.14	0.771
Osteophyte score	7.09 ± 0.09	6.78 ± 0.09	0.015	5.91 ± 0.27	6.15 ± 0.05	0.393
JSN score	7.75 ± 0.12	7.94 ± 0.11	0.243	8.93 ± 0.40	9.17 ± 0.08	0.547
Subchondral cyst score	0.64 ± 0.04	0.66 ± 0.04	0.668	1.40 ± 0.16	1.32 ± 0.03	0.622
Sclerosis score	0.54 ± 0.04	0.37 ± 0.04	0.002	0.40 ± 0.11	0.35 ± 0.02	0.592
Erosion score	0.29 ± 0.04	0.17 ± 0.04	0.027	0.41 ± 0.14	0.34 ± 0.03	0.642
Malalignment score	0.14 ± 0.02	0.08 ± 0.02	0.029	0.21 ± 0.07	0.15 ± 0.01	0.404

Unless otherwise indicated, values are mean ± standard deviation.

Data are adjusted for age, body mass index and gender in the analysis of total subjects, and are adjusted for age and body mass index in the gender stratified analysis.

One drink was assumed to contain 10 g of ethanol.

JSN, joint space narrowing.

For male participants, those who consumed one or more drinks per day had higher scores in the knee joints for total knee score (P = 0.017), osteophyte score (P = 0.029), tibial attrition score (P = 0.045), and sclerosis score (P = 0.003) compared to men who consumed less than one drink per day after adjusting for age and BMI. Additionally, in the hand joints, men who consumed one or more drinks per day also displayed higher scores in osteophyte score (P = 0.015), sclerosis score (P = 0.002), erosion score (P = 0.027), and malalignment score (P = 0.029) after adjusting for relevant factors. In contrast, among female participants, there were no statistically significant differences in the knee and hand joint scores between those who consumed one or more drinks per day and those who consumed less than one drink per day.

### Association of ALDH2 rs671 genotype with radiographic severity in the knee and hand joints

Table 4 shows the association between ALDH2 rs671 genotypes and radiographic severity in the knee and hand joints. Among all subjects, those with the G/G genotype exhibited higher scores in the knee joint for total score (P = 0.015), osteophyte score (P = 0.012), tibial attrition score (P = 0.040), and sclerosis score (P = 0.045) compared to individuals with G/A and A/A genotypes after adjusting for age, gender, and BMI. In the hand joints, subjects with the G/G genotype also displayed higher osteophyte score (P = 0.011) and sclerosis score (P = 0.019) after similar adjustments.

**Table 4:** Association between rs671 genotypes and radiographic severity scores in the knee and hand joints.

	Men			Women		
	G/G	G/A and A/A	P value	G/G	G/A and A/A	P value
Knee joints						
Total score	14.07 ± 0.20	12.78 ± 0.31	< 0.001	15.21 ± 0.21	15.08 ± 0.32	0.742
Osteophyte score	7.41 ± 0.11	6.76 ± 0.16	0.001	8.02 ± 0.02	7.88 ± 0.18	0.514
JSN score	5.87 ± 0.08	5.46 ± 0.13	0.008	6.26 ± 0.07	6.31 ± 0.11	0.688
Tibial attrition score	0.24 ± 0.02	0.16 ± 0.03	0.026	0.26 ± 0.02	0.24 ± 0.03	0.439
Sclerosis score	0.55 ± 0.03	0.40 ± 0.05	0.005	0.67 ± 0.03	0.66 ± 0.04	0.799
Hand joints						
Total score	16.41 ± 0.18	15.74 ± 0.28	0.042	17.48 ± 0.17	17.44 ± 0.26	0.900
Osteophyte score	7.00 ± 0.08	6.73 ± 0.11	0.058	6.20 ± 0.06	6.01 ± 0.10	0.086
JSN score	7.86 ± 0.10	7.84 ± 0.15	0.898	9.12 ± 0.09	9.27 ± 0.14	0.376
Subchondral cyst score	0.66 ± 0.04	0.63 ± 0.05	0.611	1.31 ± 0.04	1.36 ± 0.06	0.440
Sclerosis score	0.51 ± 0.03	0.33 ± 0.05	0.002	0.35 ± 0.02	0.34 ± 0.04	0.800
Erosion score	0.26 ± 0.03	0.15 ± 0.05	0.062	0.35 ± 0.03	0.32 ± 0.05	0.067
Malalignment score	0.12 ± 0.01	0.06 ± 0.03	0.030	0.15 ± 0.02	0.15 ± 0.02	0.919

Unless otherwise indicated, values are mean ± standard deviation.

Data are adjusted for age, body mass index and gender in the analysis of total subjects, and are adjusted for age and body mass index in the gender stratified analysis.

JSN, joint space narrowing.

For male participants, those with the G/G genotype showed higher scores in the knee joint for total score (P < 0.001), osteophyte score (P = 0.001), JSN score (P = 0.008), tibial attrition score (P = 0.026), and sclerosis score (P = 0.005) compared to individuals with G/A and A/A genotypes after adjusting for age and BMI. In the hand joints, men with the G/G genotype also demonstrated higher total score (P = 0.042), sclerosis score (P = 0.002), and malalignment score (P = 0.030) following the adjustments. However, among women, there were no statistically significant differences in the knee and hand joint scores between those with the G/G genotype and those with G/A and A/A genotypes.

### Association of alcohol drinks with radiographic severity in the knee and hand joints using MR analysis

Table 5 presents the results of the MR analysis, demonstrating the relationship between genetically predicted alcohol intake and radiographic severity in the knee and hand joints among men. In the instrumental variable analysis, for each alcohol drink per day, knee joint scores for total score, osteophyte score, JSN score, tibial attrition score, and sclerosis score increased by 0.68 (95% confidence interval [CI], 0.29–1.06; P = 0.001), 0.34 (95% CI 0.14–0.55; P = 0.001), 0.21 (95% CI 0.05–0.37; P = 0.009), 0.04 (95% CI 0.00–0.08; P = 0.028), and 0.08 (95% CI 0.02–0.14; P = 0.006), respectively, after adjusting for age and BMI. Moreover, in the hand joint scores, sclerosis score and malalignment score also increased by 0.09 (95% CI 0.03–0.15; P = 0.004) and 0.04 (95% CI 0.00–0.07; P = 0.028) for each alcohol drink per day, respectively, after the same adjustments.

**Table 5:** Association of alcohol (drinks per day) with degree of radiographic severity in the knee and hand joints in men using a Mendelian randomization analysis.

	Mendelian randomization instrumental variable analysis		Observational multivariable regression analysis	
	Beta coefficient	P value	Beta coefficient	P value
Knee joints				
Total score	0.68 (0.29–1.06)	0.001	0.21 (0.08–0.34)	0.001
Osteophyte score	0.34 (0.14–0.55)	0.001	0.11 (0.04–0.18)	0.001
JSN score	0.21 (0.05–0.37)	0.009	0.06 (0.00–0.11)	0.038
Tibial attrition score	0.04 (0.00–0.08)	0.028	0.01 (0.00–0.02)	0.096
Sclerosis score	0.08 (0.02–0.14)	0.006	0.03 (0.01–0.05)	0.001
Hand joins				
Total score	0.34 (− 0.01 to 0.69)	0.053	0.14 (0.03–0.26)	0.013
Osteophyte score	0.13 (− 0.02 to 0.27)	0.082	0.07 (0.02–0.12)	0.005
JSN score	0.01 (− 0.17 to 0.20)	0.880	0.00 (− 0.07 to 0.06)	0.895
Subchondral cyst score	0.01 (− 0.05 to 0.08)	0.684	0.00 (− 0.02 to 0.02)	0.957
Sclerosis score	0.09 (0.03–0.15)	0.004	0.05 (0.03–0.07)	< 0.001
Erosion score	0.06 (0.00–0.12)	0.071	0.02 (0.00–0.04)	0.026
Malalignment score	0.04 (0.00–0.07)	0.028	0.01 (0.00–0.02)	0.043

The values are presented as beta coefficient (95% confidence interval).

Data are adjusted for age and body mass index.

JSN, joint space narrowing.

In the observational multivariable regression analysis, each alcohol drink per day was associated with increased knee joint scores for total score (0.21 for each drink; 95% CI 0.08–0.34; P = 0.001), osteophyte score (0.11 for each drink; 95% CI 0.04–0.18; P = 0.001), JSN score (0.06 for each drink; 95% CI 0.00–0.11; P = 0.038), and sclerosis score (0.03 for each drink; 95% CI 0.01–0.05; P = 0.001) after adjustment. In the hand joint scores, each alcohol drink per day increased the scores for total score (0.14 for each drink; 95% CI 0.03–0.26; P = 0.013), osteophyte score (0.07 for each drink; 95% CI 0.02–0.12; P = 0.005), sclerosis score (0.05 for each drink; 95% CI 0.03–0.07; P < 0.001), erosion score (0.02 for each drink; 95% CI 0.00–0.04; P = 0.026), and malalignment score (0.01 for each drink; 95% CI 0.00–0.02; P = 0.043) after adjustment.

However, in women, there were no significant differences between genetically predicted alcohol intake and radiographic severity in the knee and hand joints, as observed through MR analysis and observational multivariable regression analysis (Table 6).

**Table 6:** Association of alcohol (drinks per day) with degree of radiographic severity in the knee and hand joints in women using a Mendelian randomization analysis.

	Mendelian randomization instrumental variable analysis		Observational multivariable regression analysis	
	Beta coefficient	P value	Beta coefficient	P value
Knee joints				
Total score	0.68 (− 3.44 to 4.79)	0.747	0.11 (− 0.45 to 0.68)	0.695
Osteophyte score	0.77 (− 1.56 to 3.09)	0.518	0.02 (− 0.30 to 0.34)	0.897
JSN score	− 0.30 (− 1.75 to 1.16)	0.688	0.04 (− 0.16 to 0.24)	0.679
Tibial attrition score	0.14 (− 0.22 to 0.50)	0.438	0.04 (0.00–0.09)	0.072
Sclerosis score	0.07 (− 0.51 to 0.64)	0.822	0.00 (− 0.07 to 0.08)	0.909
Hand joints				
Total score	0.17 (− 3.21 to 3.56)	0.921	− 0.22 (− 0.69 to 0.24)	0.347
Osteophyte score	1.08 (− 0.16 to 2.32)	0.089	− 0.09 (− 0.26 to 0.08)	0.313
JSN score	− 0.85 (− 2.66 to 0.96)	0.358	− 0.16 (− 0.41 to 0.09)	0.198
Subchondral cyst score	− 0.29 (− 1.01 to 0.43)	0.429	− 0.01 (− 0.11 to 0.09)	0.866
Sclerosis score	0.06 (− 0.44 to 0.56)	0.810	0.02 (− 0.04 to 0.09)	0.481
Erosion score	0.14 (− 0.49 to 0.77)	0.654	0.00 (− 0.09 to 0.09)	0.985
Malalignment score	0.02 (− 0.30 to 0.33)	0.920	0.01 (− 0.03 to 0.05)	0.675

The values are presented as beta coefficient (95% confidence interval).

Data are adjusted for age and body mass index.

JSN, joint space narrowing.

## Discussion

In this study, we have demonstrated that subjects who consumed one or more drinks per day had higher radiographic severity of knee and hand joints in total subjects and men. Similarly, subjects with G/G genotype who drank more alcohol per day had higher radiographic severity of knee and hand joints than those with G/A and A/A genotypes in total subject and men. Additionally, in the MR analysis using ALDH2 rs671 genotypes as an instrumental variable for alcohol consumption, each alcohol drink per day was significantly associated with radiographic severity of knee and hand joints, particularly in men, but not in women.

Our study is the first to establish a significant association between alcohol consumption and the radiographic severity of knee and hand joints through a MR approach. Previous observational studies have yielded varying results regarding the impact of alcohol intake on hand and knee OA. Specifically, in hand OA, participants from the Osteoarthritis Initiative study with moderate alcohol consumption showed higher K–L scores at baseline, a trajectory of increasing K–L scores over a 4-year follow-up period, and a two-fold higher likelihood of erosive hand OA^3^. In the case of knee OA, a study based on the fifth Korean National Health and Nutrition Examination Survey found a positive correlation between the degree of alcohol consumption, as measured by the Alcohol Use Disorders Identification Test score, and the degree of radiographic knee OA^8^. Conversely, a cross-sectional study conducted in Israel indicated a negative association between moderate alcohol consumption and radiographic hand OA in women, as assessed by K–L scores^4^. Furthermore, the Michigan Bone Health Study did not find any significant associations between alcohol consumption and radiographic hand and knee OA, as determined by K–L scores^7^. Notably, while monthly alcohol consumption was linked to ultrasound-detected synovitis, the frequency of alcohol consumption did not show any association with radiographic hand OA, assessed by K–L grading, in the MUST study^6^. Similarly, a cross-sectional study from Japan did not identify any association between alcohol consumption and radiographic knee OA^9^. Our results are in line with the findings from the Osteoarthritis Initiative study and the fifth Korean National Health and Nutrition Examination Survey. Several factors may account for the considerable differences in study outcomes, including variations in the number of subjects studied, the fact that alcohol intake was not the primary focus in previous studies, limitations associated with K–L grading in radiological assessments, and the evaluation of individual joints without considering the entire joint complex. In our investigation, we drew upon a sizable study population from the Dong-gu cohort, characterized by systematic data collection and radiological imaging. Unlike previous observational studies where alcohol consumption was not the primary research focus and relied on patient memory or questionnaires, our study prioritized alcohol consumption as the primary variable of interest. Additionally, the MR approach was employed to elucidate and support the causal relationship between ALDH2 rs671 genotypes and alcohol consumption. It is noteworthy that previous studies predominantly assessed OA severity through K–L grading, with only a few conducting a simultaneous analysis of knee and hand OA. By contrast, our study utilized a more comprehensive, semi-quantitative grading system to evaluate hand and knee OA concurrently, enhancing the reliability of our results and overall strength of the study.

The mechanistic pathways by which alcohol affects cartilage or chondrocytes are still not fully elucidated. However, recent research sheds light on potential mechanisms. Acute alcohol exposure induces anti-inflammatory effects in both mice and humans, potentially reducing inflammatory cytokine production^17^, whereas chronic alcohol consumption appears to trigger a systemic inflammatory response that involves an increase in the release of pro-inflammatory cytokines and a simultaneous reduction in anti-inflammatory mediators, ultimately impairing the synthesis of joint cartilage^18^. In a study using an OA-like C57BL/6 mouse model exposed to an alcohol diet containing 4.5% ethanol for 8 weeks, chronic alcohol consumption led to a significant reduction in anti-inflammatory cytokines such as TIMP-3 and SOCS-2 in articular chondrocytes, as evidenced by immunohistochemistry^19^. This reduction was associated with increased loss of cartilage proteoglycans in knee and shoulder joints. In vitro experiments using a monolayer culture of articular chondrocytes derived from patients with non-traumatic femoral head necrosis revealed that alcohol inhibited chondrocyte growth, as demonstrated by cell proliferation and viability assays^20^. Moreover, alcohol decreased the mRNA expression of cartilaginous genes such as SOX9 and aggrecan, as well as osteogenesis-related gene osteoprotegerin and the pleiotropic cytokine transforming growth factor-β, suggesting a potential acceleration of cartilage degradation. In line with these findings, a recent meta-analysis demonstrated that individuals with chronic alcohol consumption exhibited elevated levels of pro-inflammatory cytokines including TNFα, IL-6, IL-7, and IL-8 compared to non-drinkers^21^. Given that inflammation plays a pivotal role in the structural progression of OA by promoting the production of inflammatory mediators by chondrocytes and synoviocytes^22^, it is plausible that alcohol consumption contributes to cartilage damage in OA by stimulating an inflammatory response while concurrently reducing anti-inflammatory mediators.

In this study, we observed a significant association between alcohol consumption and the radiographic severity of knee and hand joints in men, but not in women. Several factors may contribute to this gender-based difference. First of all, men are more likely to drink than women^23^, the social environment generally enables women to have a healthier lifestyle^24^, and the activity of alcohol dehydrogenase in men is higher than that in women^25^. Our study confirmed these trends, with 67.3% of men being current drinkers compared to 36.0% of women. Men also consumed more alcohol per day, averaging 1.89 drinks versus women's 0.15, regardless of ALDH2 rs671 genotypes. Particularly, only 3.7% of women reported consuming one or more drinks per day. Due to the limited number of women who consumed alcohol in our study, statistical power may have been insufficient to detect significant differences in knee and hand joint radiographic severity between women who consumed alcohol and those who did not. Expanding the sample size specifically among female alcohol consumers could address this limitation and potentially yield more meaningful results. Secondly, concerning hormonal factors, a role for sex hormones in the development of OA has been suggested^26,27^. Results from many epidemiological studies show that the prevalence of OA is similar in men and women until age 50, after which it increases more rapidly in women, suggesting that estrogen deficiency may promote OA development^28^. Although controversial, estrogens and their receptors are thought to play protective roles in articular cartilage biochemistry^29,30^. Estrogens have been shown to mitigate pain in a dose-dependent manner, and testosterone reduces sensitivity to chronic pain, which could explain the accelerated progression of OA in women post-menopause^31^. Additionally, women have been observed to have smaller cartilage volumes and cartilage surface areas than men, as measured by MRI^32^, and women tend to lose cartilage volume more rapidly than men over time in symptomatic knee OA patients^33^. Our study, which targeted individuals over 50 years of age, demonstrated that women had higher radiographic scores of knee and hand joints than men. However, no association was found between alcohol consumption and the radiographic severity of knee and hand joints in women. It is possible that alcohol affects postmenopausal women differently than men, and this is unlikely to be solely explained by an estrogen effect. Further research is needed to fully understand the interplay between sex hormones, alcohol consumption, and OA severity. Thirdly, from a behavioral perspective, while a certain level of mechanical joint stress is essential for maintaining joint health, excessive stress may exacerbate OA development. Interestingly, moderate exercise appears to have a weak protective effect, potentially more so in women than in men^34^. This observation supports the hypothesis that, assuming equal levels of physical activity between genders, the activities undertaken by women might differently influence the impact of alcohol on worsening radiographic OA severity.

Our study has several strengths. Firstly, it provides evidence of a causal relationship between alcohol consumption and increased radiographic scores in knee and hand joints affected by OA through the employment of a MR instrumental variable analysis. Specifically, ALDH2 rs671 genotypes were utilized as instrumental variables for assessing the impact of alcohol intake on the radiographic characteristics of knee and hand OA. This approach mitigates common sources of error, confounding factors, and several of challenges pertaining to causality that have plagued observational studies. Secondly, our study benefits from a robust dataset derived from a substantial population-based cohort study. The substantial sample size enhances the external validity and reliability of our findings, bolstering their applicability to other populations. Thirdly, we implemented a semi-quantitative grading system to evaluate the radiographic severity of OA. This approach offers heightened sensitivity and yields continuous data, thereby affording more refined statistical analyses. This approach is particularly advantageous in uncovering nuanced trends and correlations that may elude detection when employing the categorical K–L scoring system. Lastly, our findings have important implications for public health policy and clinical practice. By understanding a patient's genetic predisposition and drinking habits, healthcare providers can offer more informed advice and interventions to potentially delay the OA progression. The significant interaction between alcohol consumption and genetic predisposition to OA severity highlights the need for genetic screening in clinical settings. Healthcare providers should consider incorporating genetic tests for ALDH2 rs671 genotypes to identify individuals at higher risk of alcohol-induced joint damage, enabling personalized and effective prevention strategies. Given the gender-specific differences in the impact of alcohol on joint health, it is crucial to develop separate guidelines for men and women regarding safe alcohol consumption levels. For men, particularly those with the G/G genotype, reducing alcohol intake may significantly lower the risk of increased joint damage. For women, although the association was not significant, moderation in alcohol consumption should still be advised. Ultimately, practicing physicians should recommend that OA patients limit their alcohol intake to mitigate the risk of increased joint damage. Policymakers should consider these findings when crafting public health guidelines and educational campaigns aimed at reducing alcohol consumption to prevent OA progression. Implementing these strategies can contribute to better management of OA and improved patient outcomes.

Nonetheless, it is imperative to acknowledge certain limitations inherent in our study. Firstly, the cross-sectional design employed in this study enables the identification of associations at a specific time point. While an MR approach is utilized, it does not establish a causal relationship or elucidate the precise mechanisms through which causality is mediated. Therefore, further research utilizing longitudinal cohorts with repeated imaging would be better suited to investigate whether alcohol consumption precedes the exacerbation of OA over time and to explore the underlying mechanisms driving the observed relationship. Secondly, it's crucial to highlight that ALDH2 rs671 genotypes primarily impact alcohol consumption among individuals who actively partake in drinking alcohol. Given the lower prevalence of alcohol consumption among women in our study cohort, the applicability of ALDH2 rs671 genotypes as a tool for measuring alcohol intake in women may be limited. Considering the potential advantages of employing genome-wide polygenic risk scores for alcohol use, rather than relying solely on single locus MR, we recognize that incorporating multiple genetic loci into a polygenic risk score could indeed enhance the effectiveness of our MR analysis in future studies. Thirdly, our study did not conduct an analysis regarding potential variations in the effects of different types of alcohol on OA. Whether different alcoholic beverages exert distinct influences on the development and progression of OA should be examined in further research. Fourthly, residual confounding effects from unmeasured variables might have biased our results. Notably, variables like physical activity, occupational hazards, nutritional factors, and history of injury, fractures, or surgery could substantially influence the risk of OA and potentially confound our study outcomes. Unfortunately, we did not collect comprehensive data on these potential confounders during our study. Future studies should aim to capture comprehensive information on these and other potential confounders to enhance the robustness of the findings through more sophisticated adjusted statistical models. Fifthly, our reliance on self-reported questionnaires for assessing alcohol intake may introduce recall bias. Employing direct quantification methods such as measuring blood alcohol levels or urine ethyl glucuronide could have offered more precise and unbiased evaluations of alcohol exposure. Sixthly, this study focused specifically on older Korean adults, which may constrain the generalizability of findings to younger age groups or individuals of non-Asian ethnicities. Replicating the study across broader geographical and demographic spectra would enhance the representation of the population. Lastly, expanding the research to include joints beyond the hands and knees and utilizing advanced imaging techniques such as MRI in addition to simple radiography would provide a more comprehensive characterization of the systemic effects of alcohol on joints. Incorporating these aspects into future studies would strengthen the conclusions regarding the impact of alcohol consumption on joint health.

## Conclusion

Our study has identified a significant link between alcohol consumption and the radiographic severity of OA in knee and hand joints. Individuals with the G/G genotype who consume more alcohol daily show increased joint severity compared to those with G/A and A/A genotypes, particularly among men. Using MR analysis with ALDH2 rs671 genotypes as instrumental variables, we found that each additional daily alcoholic drink correlates with greater joint severity in men, but not in women. These findings underscore the gender-specific effects of alcohol on joint health, suggesting that reducing alcohol consumption could help prevent OA progression.

## Data Availability

Full original protocol and dataset can be accessed upon request for academic researchers by contacting Professor Shin-Seok Lee (shinseok@chonnam.ac.kr).
